# Epicellular Apicomplexans: Parasites “On the Way In”

**DOI:** 10.1371/journal.ppat.1005080

**Published:** 2015-09-24

**Authors:** Pavla Bartošová-Sojková, Rebecca D. Oppenheim, Dominique Soldati-Favre, Julius Lukeš

**Affiliations:** 1 Institute of Parasitology, Biology Centre, Czech Academy of Sciences, České Budějovice (Budweis), Czech Republic; 2 Department of Microbiology and Molecular Medicine, University of Geneva Medical School, Geneva, Switzerland; 3 Canadian Institute for Advance Research, Toronto, Ontario, Canada; 4 Faculty of Science, University of South Bohemia, České Budějovice (Budweis), Czech Republic; University of Wisconsin Medical School, UNITED STATES

## Apicomplexans from Cold-Blooded Vertebrates

The Coccidia and the Cryptosporidia infect both cold- and warm-blooded vertebrates, yet members of the genera *Toxoplasma*, *Eimeria*, *Sarcocystis*, and *Cryptosporidium* (which mostly parasitize the latter hosts) have received most of the attention by far because of their importance to human and veterinary health. Our knowledge about a wide array of apicomplexans found in fish, amphibians, and reptiles is thus primarily confined to the morphological description of their exogenous stages (i.e., oocysts) and sites of infection, rarely with notes on pathogenicity. The life cycles of these “neglected” parasites resemble those of apicomplexans from birds and mammals. They undergo successive multiplication by merogony (asexual divisions producing meronts with merozoites), followed by gamogony (fusion of two types of gametes resulting in an oocyst), and sporogony (asexual reproduction producing sporozoites) [1]. In the Coccidia of homeotherms, these developmental phases take place in different hosts, whereas the majority of the Coccidia of poikilotherms typically have direct life cycles. The intestine is the favored site for sporogony, but piscine coccidians are characterized by their frequent extraintestinal development with either exogenous (outside the host) or endogenous (inside the host) sporulation [1]. Coccidians of poikilotherm hosts differ from those parasitizing homeotherms by (1) thin-walled oocysts lacking micropyle (an opening of oocyst wall), (2) macrogamonts without wall-forming bodies, (3) sporocysts with excystation structures (sutures) and projections of the sporocyst wall (membranaceous veils, sporopodia), and (4) frequent epicellular localization, which is shared with some gregarines, the protococcidian *Eleuteroschizon dubosqi*, and cryptosporidians [1–4].

**Fig 1:**
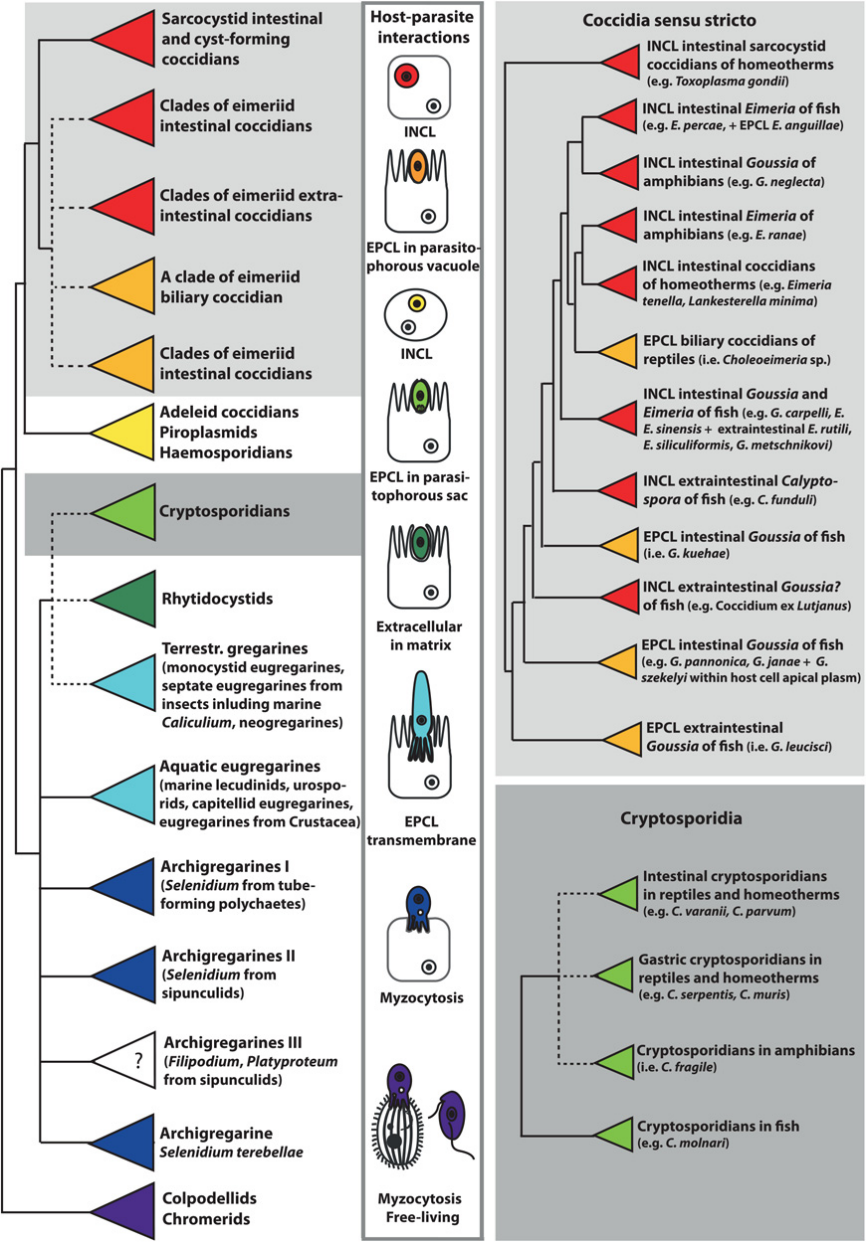
This schematic phylogenetic tree shows the relationships among major groups of Apicomplexa and the evolution of host–parasite interactions. Phylogenetic analysis is based on the alignment of 18S rRNA gene sequences from 140 apicomplexan taxa, which were aligned in Multiple Alignment using Fast Fourier Transform (MAFFT) and analyzed using maximum likelihood criterion in RAxML, both implemented in Geneious version 8.1.3. Coccidians are monophyletic, with epicellular (EPCL) species positioned at the base of the eimeriid group and the cryptosporidia cluster within gregarines. The parasitic lifestyle likely evolved from the free-living chromerids via the myzocytic feeding of colpodellids and archigregarines. The feeding strategies of *Platyproteum* and *Filipodium* (“archigregarine” clade III) are unknown (labelled with question mark), but most likely include a kind of surface-mediated acquisition of nutrients. Neogregarines and eugregarines developed a more permanent transmembrane feeding, whereas cryptosporidians exhibit EPCL mode of nutrition using the elaborate feeder organelle. EPCL coccidians uptake nutrients through the microvillous layer of host cells. Intracellular (INCL) Apicomplexa (most eimeriid and sarcocystid coccidians, piroplasms, and haemosporidians) use both transmembrane and micropore modes of nutrient uptake. Micropores are also used for endocytosis in rhytidocystids, which are embedded in the extracellular matrix of the host’s intestinal epithelium.

Phylogenetic analyses based on the 18S rRNA gene sequences, now available from a number of apicomplexans parasitizing cold-blooded vertebrates (e.g., members of the genera *Cryptosporidium*, *Goussia*, *Acroeimeria*, *Eimeria*, *Calyptospora*, and *Choleoeimeria*), allowed for the inference of their phylogenetic relationships. Piscine cryptosporidians represent a well-defined monophyletic group at the base of the cryptosporidian clade, whereas amphibian and reptile cryptosporidians are mixed with those infecting homeotherms. Coccidians of poikilotherm hosts constitute basal lineages of the whole eucoccidian clade, or of its eimeriid or sarcocystid subclades, with piscine coccidians representing the most basal groups (Fig 1) [2,3,5–7]. Because of their early-branching position, these parasites of poikilotherms likely possess ancestral features, the scrutiny of which may help us better understand the evolution of Apicomplexa. Indeed, the simple excystation structures of piscine, amphibian, and reptile coccidians have diverged into more complex excystation structures in eimeriids, sarcocystids, and calyptosporiids [2,3]. The same may apply for the site of infection, as the epicellular development seems to have been abandoned during the evolution of mammalian and avian coccidians.

Herein, we review the modes of interactions and invasion mechanisms of the epicellular apicomplexans with their cold-blooded hosts. Furthermore, we discuss host cell invasion and metabolic adaptations, as well as map characters of host–parasite interactions on the 18S rRNA gene-based schematic phylogenetic tree (Fig 1), with a special focus on coccidians and cryptosporidians from poikiloterm hosts.

## An Overlooked Spectrum of Host–Parasite Physical Interactions

Merozoites of the epicellular species infecting fish, amphibians, and reptiles exhibit all the typical attributes of apicomplexans, yet they reside in a host-derived envelope that adopts diverse morphologies; consequently, these parasites likely rely on various modes of nutrient uptake. Cryptosporidians reside in an incomplete parasitophorous sac (PS), as they are directly connected to the host stomach or enterocyte epithelial membrane via a multimembrane feeder organelle (MFO) [8]. On the other hand, in the epicellular (also known as extracytoplasmic) Coccidia, this envelope (termed parasitophorous vacuole [PV]) fully encloses the parasite, and the PV membrane (PVM) associates with the host cell cytoplasm, either in one large smooth area or by forming various projections, folds, spines, and invaginations, most probably enhancing acquisition of nutrients and/or attachment to the host cell. In some epicellular Coccidia, the PV lumen contains high levels of calcium and phosphate ions, probably reflecting its metabolic activity [9]. On the lumen-facing side, two tightly apposed host membranes cover the parasite: the outer one being the enterocyte or kidney epithelial cell membrane and the inner one the PVM [6,9–15].

**Fig 2:**
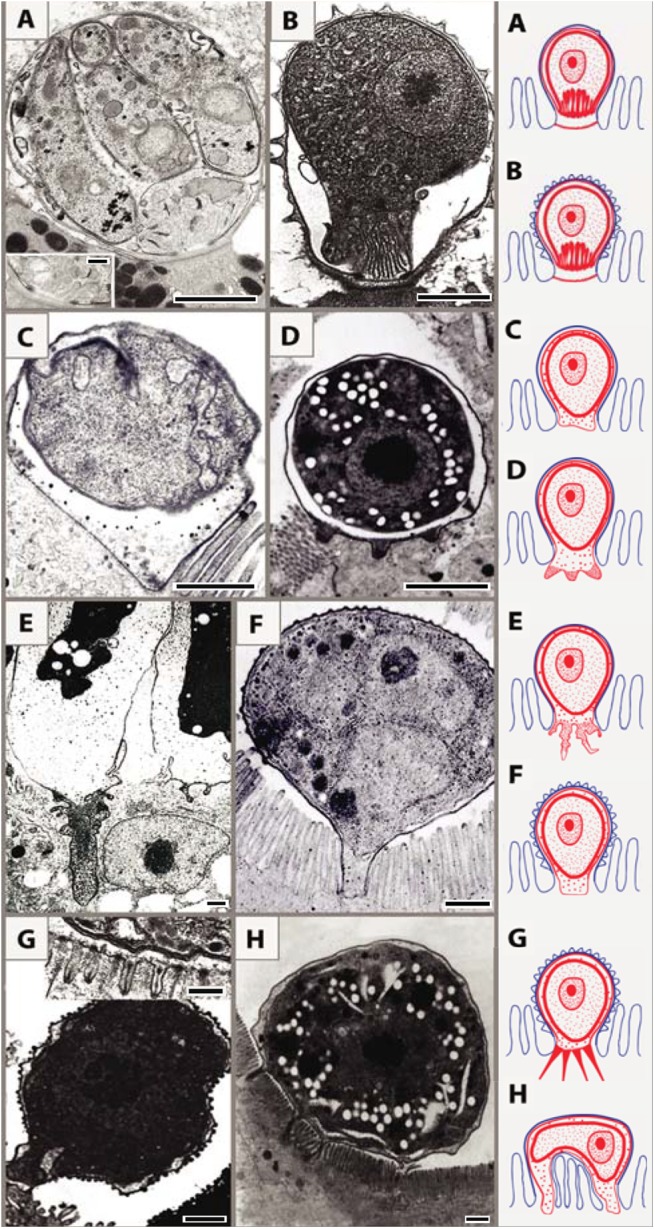
Schematic interpretation of host–parasite interactions of epicellular piscine cryptosporidians and coccidians. Monopodial stage of *Cryptosporidium molnari* with the detail of feeder organelle (inset) (A; modified from Alvarez-Pellitero P, Palenzuela O, Sitjà-Bobadilla A. 2002. *Cryptosporidium molnari* n. sp. (Apicomplexa: Cryptosporidiidae) infecting two marine fish species, *Sparus aurata* L. and *Dicentrarchus labrax* L. Figs 23 and 30. International Journal for Parasitology 32: 1007–1021. Elsevier 2002), *Cryptosporidium villithecus* (B; adapted from Landsberg and Paperna, 1986), *Goussia janae* (C; adapted from Lukeš J, Starý V. 1992. Ultrastructure of life cycle stages of *Goussia janae* (Apicomplexa, Eimeriidae) and X-ray microanalysis of accompanying precipitates. Canadian Journal of Zoology, 70 (12): 2382–2397, 2008 Canadian Science Publishing or its licensors. Reproduced with permission), *Eimeria anguillae* (D), *Goussia spraguei* (E; adapted from A new species of *Goussia* (Apicomplexa, Coccidia) in the kidney tubules of the cod, *Gadus morhua* L. Morrison CM, Poynton SL. Journal of Fish Diseases 12 (6). 1989. John Wiley & Sons, Inc. http://onlinelibrary.wiley.com/doi/10.1111/j.1365-2761.1989.tb00564.x/abstract), *Eimeria puytoraci* (F), *Eimeria vanasi* with the detail of its protrusions into the host cell cytoplasm (inset) (G; adapted from Paperna, (1991). Kim and Paperna (1992)); Spider-like stage of *Goussia pannonica* (H; adapted from Life cycle of *Goussia pannonica* (Molnár, 1989) (Apicomplexa, Eimeriorina), an extracytoplasmic coccidium from the white bream *Blicca bjoerkna*. Lukeš J. Journal of Protozoology 39 (4). 1992 John Wiley & Sons, Inc. http://onlinelibrary.wiley.com/doi/10.1111/j.1550-7408.1992.tb04836.x/abstract). In all figures, the scale bar is 1 μm, with the exception of insets (0.2 μm in A and 0.3 μm in G).

Based on ultrastructural features, we distinguish eight types of host–parasite interfaces among epicellular species from fish and reptiles that fit into the monopodial (single attachment zone; 1–7) types or multipodial (8) types: (1) MFO in the contact zone with smooth outer host cell membrane (Fig 2A) [10]; (2) MFO in the contact zone with microvillous surface of the outer host cell membrane (Fig 2B) [11]; (3) smooth contact zone without any protrusions (Fig 2C) [9]; (4) regular finger-like protrusions of the PV into the host cytoplasm, filled with globular granules (Fig 2D) (Lukeš J, unpublished results); (5) deep irregular invaginations of the PV filled with dense granular material (Fig 2E) [12]; (6) smooth interface with abundant granular, material-containing verrucous protuberances on the outer enterocyte membrane (Fig 2F); (7) numerous long tubular formations extending from the PV, which carries small projections or ridges on its luminal side (Fig 2G) [13,14]; (8) spider-like stages localized above the microvillar region that are connected with the host cytoplasm via several projections (Fig 2H) [15].

**Fig 3:**
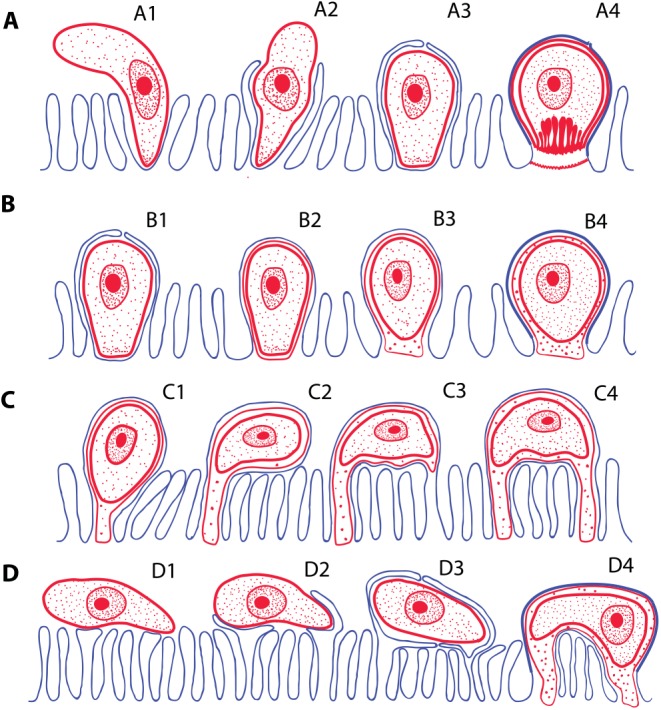
Schematic interpretation of mechanisms of host invasion by the epicellular (EPCL) piscine cryptosporidians and coccidians. (**A**) Invasion of host cell by *Cryptosporidium* spp. Zoite penetrates among the microvilli (A1), which eventually surround and enclose it within the parasitophorous sac (A2–A3). Finally, the feeder organelle is formed (A4). (**B)** The invasion mechanism resulting in the formation of monopodial stages of an EPCL coccidium. The beginning of invasion, until the enclosure of the parasite by the host cell membrane, is similar as in A (B1), but the feeder organelle does not form (B2) and the attachment remains monopodial (B3–B4). (**C** and **D)** Two possible mechanisms of formation of the spider-like EPCL stages. (**C**) The zoite settles down and fuses with the apical part of the microvillus (C1) and its growth progressively extends to the neighbouring microvilli (C2). Contact between the infected microvillar membrane and the membranes of other microvilli (C3) results in their fusions (C4), thus attaching the parasite to the host cell via additional region(s). (**D)** The zoite settles down at the microvillar zone laterally (D1), inducing fusion of non-neighbouring microvilli and its enclosure (D2–D3), thus forming a spider-like stage attached to the host cell (or more cells) in several regions (D4).

Following the penetration of the cryptosporidian zoite among the microvilli (Fig 3A1), cytoplasmic extensions of the host cell membrane start surrounding it (Fig 3A2) and eventually enclose the zoite in a PS (Fig 3A3 and 3A4) [8]. In the epicellular Coccidia, the infection starts with a contact between apical part of the motile zoite and glycocalyx of the enterocyte microvilli. The recognition phase is followed by an invasion of the enterocyte and formation of monopodial or spider-like stages. The initial contact causes hypertrophy of the surrounding microvilli and their subsequent fusion, resulting in the enclosure of the parasite in the microvillar zone (Fig 3B1–3B4). Growth of the coccidium leads to additional lateral fusions and expansion of the contact area. The spider-like stages establish themselves via multiple fusions with several non-neighbouring microvilli (Fig 3C and 3D). It appears that the epicellular Coccidia trigger the formation of small undulations of the host cell membrane, which progressively turn into projections, the tips of which fuse with adjacent microvilli (Fig 2H). In the next phase, the PVM passes through the lumen of a newly fused microvillus until it reaches the host cytoplasm, thus establishing a new host–parasite interface. Through multiple projections, the PVM becomes connected with the cytoplasm of a single host cell or even with several cells.

## RONs-AMAs: A Molecular Tool for Parasites “On the Way In?”

The mechanism and the associated molecular factors allowing apicomplexans to progress from an epicellular to a complete intracellular invasion process are currently unknown. Host cell entry is a highly organized multi-step process whereby the parasite first recognizes and attaches to the host cell via the secretion of microneme adhesins. Importantly, an apical membrane antigen (AMA) family member [16] and several rhoptry neck (RON) proteins discharged by the rhoptries that act as anchoring factors into the host cell cortical cytoskeleton participate in the formation of a junction through which the parasite squeezes to access the host cell [17]. Interestingly, *Cryptosporidium*, which does not penetrate deeply into the host cytoplasm but remains epicellular, lacks AMAs and RONs that are necessary to form such a junction. In contrast, despite being epicellular, *Goussia janae*, which is able to form protrusions of the PVM and position itself deeper into the host cytoplasm, also possesses the AMAs and RON proteins [6].

## Adaptation of Nutrient Acquisition Mechanisms throughout Evolution

Myzocytosis was most probably the mode of nutrition used by the common myzozoic ancestor, as this feeding behaviour is still retained in early-diverging dinoflagellates, syndinians, perkinsids, and colpodellids (Fig 1) [18]. During myzocytosis, the parasite pierces the host cell with a feeding tube (pseudoconoid), sucking out the cellular content and digesting it. Within the Apicomplexa, the intimate association between archigregarines and their marine invertebrate hosts seems to retain features of this micropredatory feeding behaviour. The eugregarines and neogregarines infecting marine and terrestrial invertebrates have expanded their host association into a permanent transmembrane attachment, sometimes with partial or complete intracellular localization.

The common ancestry of cryptosporidians and gregarines [18] is exemplified by the attachment strategy of the protococcidian *Eleutheroschizon duboscqi*, conspicuously resembling an epicellularly located gregarine with its PS developing in a cryptosporidian manner [4]. While the exact phylogenetic relationships between cryptosporidians and particular gregarine lineage(s) have yet to be resolved, *Rhytidocystis* probably represents an evolutionary link between these groups [19]. In Cryptosporidia located on the microvillous surface of the host epithelium, nutrient uptake apparently occurs through the MFO and/or via transmembrane transporters [20,21].

The evolution of host–parasite interactions in the Coccidia may be the result of exploitation of the host. After invasion, the primitive coccidian may have attached to the gut epithelium (epicellular species), later penetrated it (intracellular intestinal species), and finally reached the internal organs via the blood and/or lymphatic system (intracellular extraintestinal species). Assuming host–parasite co-evolution, the gut became the primary site for the intracellular development after vertebrates moved from aquatic to terrestrial habitats. Since the intestine allows better spreading of oocysts than the spleen, liver, and other organs, the eimeriids and sarcocystids preferentially adopted this localization.

As reported here, the epicellular development appears to have evolved independently in gregarines, cryptosporidians, and some coccidians as an analogous adaptation to a similar environment within the host (Fig 1). Advantages of the epicellular localization may include avoidance of the immune response and uptake of solute transport across the PVM from the host cytoplasm. Epicellular parasites seem to cause only very limited damage to their host, as the infected epithelium is readily sloughed off and replaced. In contrast to cryptosporidians, the epicellular coccidians of cold-blooded vertebrates appear to be exploratory and/or flexible, as they developed numerous types of PVM projections (Figs 2 and 3) but also learned how to manipulate host membranes, as is apparent from the induced fusions of host–parasite membranes [9]. Such a behaviour requires an elaborated subversion mechanism, probably involving the insertion of parasite effector molecules into both PVM [22] and host cell membranes [23]. The recently identified dense granules (GRAs) that function as effectors in subverting host cell function during *Toxoplasma gondii* tachyzoites infection [24] cannot be found in *G*. *janae*. However, the aspartyl protease 5 involved in their processing and export beyond the PVM is present, which suggests that species-specific effectors likely exist in piscine coccidians [6].

## Flexible Metabolic Pathways in Apicomplexa

As previously described, the switch from a free-living to an obligate intracellular lifestyle is represented by the formation of specific parasitic niches and the evolution of very diverse nutrient acquisition behaviours as the parasite made its way into the host cell. This is differentially achieved in apicomplexans by species-specific adjustments of the core calcium-regulated machinery and secretory organelles [25]. With the expansion of the-omics approaches, comparative genomics and metabolomics can be applied among the epicellular and intracellular apicomplexans, and free-living dinoflagellates. There is increasing evidence that the evolution of the metabolic capabilities, originally thought to have driven the switch from free-living to intracellular parasitism, actually correlates better with specific nutrient availability in the host niche that the parasite encounters, rather than with its lifestyle [6,26]. Indeed, due to its exposure to nutritionally different environments, parasites often dispense of or inhibit entire core metabolic pathways and sometimes even lose metabolically active organelles, consequently relying on the host even more for metabolite acquisition and survival (e.g., cryptosporidians and gregarines have lost their relict plastid called the apicoplast, which in other apicomplexans houses important metabolic pathways such as part of the heme biosynthesis, fatty acid synthesis of type II, and the 1-deoxy-d-xylulose-5-phosphate [DOXP] pathway).

This versatility can be clearly exemplified by the metabolic flexibility of the epicellular Cryptosporidia. Indeed, intestinal human parasites *Cryptosporidium parvum* and *C*. *hominis* have undergone reductive evolution of their mitochondrion, leading to the loss of organellar DNA and several key functions (such as the tricarboxylic acid [TCA] cycle and respiratory chain), whereas the closely related gastric parasite of rodents, *C*. *muris*, has retained all enzymes of the TCA cycle and most complexes of the respiratory chain [27].

The first RNA-seq analyses of the *G*. *janae* oocyst/sporozoite stages provide evidence that this coccidium possesses enzymes implicated in most of the central carbon metabolism [6], resembling the versatile metabolic capabilities of *T*. *gondii* rather than the reduced ones of *Cryptosporidium* spp. which, however, share with *G*. *janae* epicellular localization. These examples highlight potential differences in the nutrients available in each niche and specific adaptions of the parasite to thrive in its environment.

In conclusion, the apicomplexans exhibit a remarkable diversity in the strategies they use for acquisition of nutrients, invasion, and interaction with host cells. From the myzocytotic ancestor, the epicellular parasitism seems to have convergently evolved in (1) gregarines + poikilotherm and homeotherm cryptosporidians and in (2) piscine + reptile coccidians, as an adaptation to colonization of different cell types and tissues. Inclusion of the epicellular apicomplexans into comparative genomic analyses, in close future, holds a promise of identifying previously unknown pathways by which these protists execute their manipulative tricks—by subversions of host cellular functions and by metabolic adaptations to the environments they encountered.
